# Association Between Perceived Quality of Postoperative Pain Care and Patient‐Reported Outcomes After Total Knee or Total Hip Arthroplasty: A Cross‐Sectional Study

**DOI:** 10.1111/jocn.70443

**Published:** 2026-07-11

**Authors:** Serdar Sever, Dilan Temir, Elif Nazlı Çelen, Sümeyra Karaduman

**Affiliations:** ^1^ Faculty of Health Sciences, Nursing Department Usak University Usak Türkiye

**Keywords:** nursing care, orthopaedic procedures, pain management, patient satisfaction, postoperative pain

## Abstract

**Aim:**

This study aimed to examine the relationship between perceived quality of postoperative pain care and patient‐reported pain‐related outcomes in patients undergoing total knee or total hip arthroplasty and to identify factors associated with higher perceived care quality.

**Design:**

A descriptive, cross‐sectional study.

**Methods:**

This study was conducted with 120 adult patients who underwent total knee or total hip arthroplasty in the orthopaedic and traumatology units of a public hospital in western Türkiye between 05 April and 30 August 2024. Data were collected using a patient information form, the American Pain Society Patient Outcome Questionnaire‐Revised (APS‐POQ‐R) and the Strategic and Clinical Quality Indicators in Postoperative Pain Management Questionnaire (SCQIPP). The SCQIPP was used as the main outcome measure, with higher scores indicating better perceived quality of postoperative pain care. Data were analysed using descriptive statistics, independent samples *t*‐tests, Pearson's correlation analysis and multiple linear regression.

**Results:**

The sample included 120 patients, of whom 98 underwent total knee arthroplasty and 22 underwent total hip arthroplasty. The mean SCQIPP score was 50.77 ± 7.41, indicating a moderate‐to‐high perceived quality of postoperative pain care. Female patients and those undergoing total knee arthroplasty surgery reported significantly higher SCQIPP scores. Higher SCQIPP scores were also observed among patients who received information about pain management and those who used non‐pharmacological pain management methods. Among APS‐POQ‐R subscales, only perception of care was significantly correlated with SCQIPP scores. In multiple regression analysis, perception of care and perceived usefulness of pain‐related information were independently associated with higher SCQIPP scores.

**Conclusion:**

The perceived quality of postoperative pain care was associated with patients' perception of care and the perceived usefulness of information about pain treatment. These findings suggest that improving postoperative pain management requires not only symptom control but also patient‐centred communication, effective education, and support for non‐pharmacological strategies.

**Implications for the Profession and/or Patient Care:**

The findings indicate that the perceived quality of postoperative pain care is shaped more by patients' experience of care, especially communication, responsiveness and the usefulness of pain‐related information, than by symptom burden alone. For nursing practice, this highlights the importance of patient‐centred pain management that combines pharmacological treatment with clear education, supportive communication and guidance on non‐pharmacological strategies. Nurses and multidisciplinary teams should not only assess pain intensity, but also evaluate whether patients feel informed, involved and supported during the postoperative period. Strengthening these aspects of care may improve patients' evaluations of pain management and contribute to a better early recovery experience after total knee and total hip arthroplasty.

**Reporting Method:**

This study was reported in line with the Strengthening the Reporting of Observational Studies in Epidemiology (STROBE) guidelines.

**Patient or Public Contribution:**

Patients contributed to the study by completing the questionnaires at hospital. However, neither patients nor members of the public were involved in the study design, data analysis, interpretation of the findings or preparation of the manuscript.

## Impact

- What Problem Did the Study Address?
  ○ This study addressed the limited evidence on how the perceived quality of postoperative pain care is associated with patient‐reported pain‐related outcomes in patients undergoing total knee or total hip arthroplasty. Although postoperative pain after total hip and knee arthroplasty remains a major clinical issue, less is known about whether patients' ratings of care quality are associated primarily with symptom severity or with how care is communicated and delivered.
- What Were the Main Findings?
  ○ The main findings were that perceived quality of postoperative pain care was significantly associated with the perception of care dimension of the APS‐POQ‐R, but not with pain severity, activity interference, affective responses or adverse effects. In multivariable analysis, perception of care and the perceived usefulness of pain‐related information were the only independent determinants of higher SCQIPP scores. Higher SCQIPP scores were also reported by female patients, patients undergoing total knee arthroplasty, those who had received information about pain management, and those using non‐pharmacological pain management methods.
- Where and on Whom Will the Research Have an Impact?
  ○ This study is likely to have an impact on nurses, orthopaedic teams and multidisciplinary professionals involved in perioperative and postoperative care, particularly in hospital settings managing arthroplasty patients. Its main impact will be on adult patients undergoing major orthopaedic surgery, especially total hip and knee arthroplasty, by informing more patient‐centred approaches to pain management. The findings support improvements in nursing assessment, education, communication and the integration of non‐pharmacological pain strategies in early postoperative care.
- What Does This Paper Contribute to the Wider Global Clinical Community?
  ○ Identifies perception of care and the perceived usefulness of pain‐related information as important factors associated with higher perceived quality of postoperative pain care after total knee and total hip arthroplasty.
  ○ Highlights that patients' assessments of pain care quality are shaped more by communication, responsiveness and supportive care processes than by pain symptoms alone.
  ○ Supports the integration of patient‐centred education, effective communication and non‐pharmacological pain management strategies into routine orthopaedic nursing practice to improve the postoperative care experience.

## 1 Introduction

Total hip arthroplasty (THA) and total knee arthroplasty (TKA) are two of the most commonly performed surgeries worldwide, and the number of patients undergoing these procedures continues to increase (Carender et al. 2025; OECD 2024). As the number of arthroplasty procedures becomes more common (Stubnya et al. 2025; Shichman et al. 2023), ensuring high‐quality postoperative care, particularly pain management, becomes increasingly important. Despite advances in surgical technique, anaesthetic practice and enhanced recovery pathways, postoperative pain after major joint replacement remains an important clinical problem (Zaslansky et al. 2022; Zhao et al. 2024).

Pain after THA and TKA is often severe, particularly in the first few days following surgery (Anger et al. 2021; Lavand'homme et al. 2022). Contemporary evidence shows that acute pain after arthroplasty remains common and a clinically important minority of patients develop persistent postsurgical pain (Cheng et al. 2025). A recent updated systematic review found that approximately 22% of patients report pain 3 months after total knee replacement, 12%–15% continue to report long‐term pain up to 2 years after TKA, and at least 14% report pain 6–12 months after THA (Cheng et al. 2025). Poorly controlled postoperative pain can interfere with early mobilization, sleep, emotional well‐being, rehabilitation participation and overall recovery (Zaslansky et al. 2022; Liu et al. 2023). In arthroplasty populations, pain trajectories also vary considerably across patients, suggesting that recovery is influenced not only by the procedure itself but also by how pain is assessed, managed and experienced in the early postoperative period (Singh et al. 2019; Omran et al. 2024).

Nurses play a key role in postoperative pain management through pain assessment, monitoring, patient education, communication and the implementation of pharmacological and non‐pharmacological interventions (Fontes et al. 2024; Youngcharoen and Aree‐Ue 2023). A recent scoping review of orthopaedic surgical patients found that quality nursing practices in pain management include the use of pain assessment scales, patient education, non‐pharmacological strategies, monitoring and staff competency development (Fontes et al. 2024). In addition, patient‐centred processes such as education and involvement in treatment decisions may influence pain‐related outcomes (Wang et al. 2024; Chen et al. 2024). Registry‐based orthopaedic studies have shown that patient participation in pain treatment decisions is associated with better patient‐reported outcomes in domains such as pain intensity, sleep interference, emotional problems and satisfaction (Jiang et al. 2022). International registry data also indicate that patient‐reported outcomes and perceptions of care provide important information that may not be captured by pain scores alone (Zaslansky et al. 2022). Therefore, evaluation of postoperative pain management should consider both symptom burden and patients' appraisals of care.

Previous studies in orthopaedic surgical populations suggest that postoperative pain outcomes are closely associated with the way pain care is delivered and perceived by patients. In patients undergoing orthopaedic surgery, Özdemir et al. (2022) reported that the quality of postoperative pain care was associated with patient care outcomes, indicating that pain management should be evaluated not only through pain intensity but also through patients' perceptions of care quality (Özdemir et al. 2022). Similarly, recent evidence from orthopaedic surgery patients has shown that satisfaction with postoperative pain management is influenced by factors such as information about pain treatment, perceived pain relief and the use of non‐pharmacological pain management methods (Khalil et al. 2025). Other studies have also highlighted that patient education, participation in pain treatment decisions and nursing responsiveness are important components of postoperative pain care in orthopaedic settings (Fontes et al. 2024; Chen et al. 2024; Jiang et al. 2022). However, less is known about which specific patient‐reported pain‐related outcome domains, such as pain severity, activity interference, affective responses, adverse effects and perception of care, are most strongly associated with perceived quality of postoperative pain care among patients undergoing THA or TKA. This is especially relevant for nursing practice, where patient education and supportive communication are modifiable aspects of care (Grommi et al. 2023; Chen et al. 2025).

Therefore, this study aimed to examine the relationship between the perceived quality of postoperative pain care and patient‐reported pain‐related outcomes among patients undergoing THA or TKA, and to identify factors associated with higher perceived quality of postoperative pain care. The study addressed the following research questions: (1) What is the perceived quality of postoperative pain care among patients undergoing total hip or total knee arthroplasty? (2) What are the patient‐reported pain‐related outcomes in this population? (3) Which patient‐reported pain‐related outcomes are associated with perceived quality of postoperative pain care? (4) Which sociodemographic, clinical and pain‐management‐related factors are independently associated with perceived quality of postoperative pain care?

## 2 Methods

This study was designed and reported in accordance with the Strengthening the Reporting of Observational Studies in Epidemiology (STROBE) guidelines (Von Elm et al. 2007).

### 2.1 Study Design, Setting, and Sample

This descriptive correlational study examined the relationship between perceived quality of postoperative pain care and patient‐reported pain outcomes among patients hospitalized in orthopaedic and traumatology wards following total knee and total hip arthroplasty. Data were collected between 05 April and 30 August 2024 in the orthopaedic and traumatology wards of a public hospital in western Türkiye. The study population comprised patients who had undergone THA or TKA, and those who met the inclusion criteria and provided informed consent were enrolled. Sample size estimation was performed using G*Power version 3.1.9.7. The calculation was based on the primary relationship of interest, namely the correlation between perceived quality of postoperative pain care measured by the SCQIPP and patient‐reported pain‐related outcomes measured by the APS‐POQ‐R. A moderate correlation coefficient of ρ = 0.30 was assumed because previous studies examining patient‐reported pain outcomes and quality or satisfaction with postoperative pain management have generally reported small‐to‐moderate associations, and 0.30 is commonly accepted as a moderate effect size in behavioural and clinical research (Brydges 2019). With a two‐tailed significance level of 0.05% and 95% statistical power, the minimum required sample size was 111 participants (Kang 2021). To account for possible incomplete data, 120 patients were included in the final analysis.

Eligible participants were adults aged 18 years and older who had undergone major orthopaedic surgery, defined in this study as total hip arthroplasty or total knee arthroplasty requiring postoperative hospitalization in the orthopaedic and traumatology ward. Patients were excluded if they had a documented psychiatric condition that could impair questionnaire completion, clinically documented dementia or cognitive impairment, or communication difficulties that prevented reliable self‐report. Communication difficulties included severe hearing impairment without adequate communication support, aphasia, inability to understand Turkish or other conditions that prevented completion of the questionnaires. Patients who required postoperative care in an intensive care unit were also excluded because their postoperative pain care pathway differed from ward‐based arthroplasty care. During the data collection period, patients who met the eligibility criteria and were available during the researcher's ward visits were invited to participate using consecutive convenience sampling. However, the total number of patients screened, the number who declined participation and reasons for non‐participation were not systematically recorded.

### 2.2 Data Collection Tools

Data were gathered using a researcher‐developed demographic and clinical information form informed by the literature (Özdemir et al. 2022; Akire and Bayraktar 2024). In addition, the American Pain Society Patient Outcome Questionnaire (APS‐POQ‐R) (Gordon et al. 2010; Keskin et al. 2019) and the Strategic and Clinical Quality Indicators in Postoperative Pain Management Questionnaire (SCQIPP) were administered (Idvall et al. 2002; Vatansever and Akansel 2014).

#### 2.2.1 Patient Information Form

Based on the literature, a patient information form was developed to collect sociodemographic and clinical data, including age, sex, body mass index, surgical procedure, type of surgery, history of previous surgery, receipt of pain‐management information, use of non‐pharmacological pain management methods and initial and current pain scores (Sınmaz and Akansel 2021; Sivaganesan et al. 2020; Karaca and Durna 2019; Hamilton et al. 2013).

#### 2.2.2 The American Pain Society Patient Outcome Questionnaire (APS‐ POQ‐R)

The APS‐POQ‐R comprises 21 items (Items 1–3, 4a–d, 5a–d, 6a–d and 7–12) organized into five subscales: (1) pain severity and sleep interference (five items; Items 1–3 and 4c–d); (2) activity interference (two items; Items 4a–b); (3) affective responses (four items; Items 5a–d); (4) adverse effects (four items; Items 6a–d); and (5) perception of care (three items; Items 7–9). The remaining three items (Items 10–12) assess patients' experiences with pain management education and nonpharmacological strategies. Specifically, Item 10 asks whether patients received information about pain treatment options, Item 11 addresses the use of nonpharmacological methods, and Item 12 examines whether healthcare professionals encouraged such methods. These items are not included in the subscale scoring (Gordon et al. 2010).

APS‐POQ‐R items are rated using 0–10 numerical response scales; however, the anchors differ according to item content. For example, pain intensity items range from no pain to worst possible pain, whereas other items assess the degree of interference, emotional response, adverse effects or perceived care. Subscale scores were calculated by summing the relevant items within each domain. A total APS‐POQ‐R score was not used in the main analysis because the instrument assesses multiple dimensions of pain‐related outcomes rather than a single unidimensional construct (Gordon et al. 2010). The Turkish adaptation of the 21‐item APS‐POQ‐R demonstrated excellent internal consistency in the 2017 validity and reliability study, with a Cronbach's alpha coefficient of 0.91 (Keskin et al. 2019). In the present study, the scale also showed satisfactory reliability, yielding a Cronbach's alpha coefficient of 0.75.

#### 2.2.3 Strategic and Clinical Quality Indicators in Postoperative Pain Management Questionnaire (SCQIPP)

The SCQIPP was used as the main outcome measure in this study. It was developed to evaluate patients' perceptions of postoperative pain management quality. The scale's validity and reliability for the Turkish population were confirmed in 2014, and it comprises 14 items (Vatansever and Akansel 2014). Each item is scored from 1 to 5, and the total score is obtained by summing all items, with possible scores ranging from 14 to 70. Higher scores indicate better perceived quality of postoperative pain care (Idvall et al. 2002). In the present study, the total SCQIPP score was used in the statistical analyses. In prior research, the SCQIPP demonstrated good internal consistency, with a Cronbach's alpha coefficient of 0.81 (Vatansever and Akansel 2014); in the present study, the scale maintained acceptable reliability, yielding a Cronbach's alpha coefficient of 0.71.

### 2.3 Data Collection Process

Data were collected 24–48 h after surgery because this period represents the early postoperative phase in which patients are usually clinically stable, have already received postoperative pain management and are able to evaluate their pain care experience. This timeframe also reduces the risk of collecting data too early, before patients have had sufficient exposure to ward‐based pain management or too late, when recall may be affected by discharge preparation. The APS‐POQ‐R was originally designed to assess pain management outcomes in hospitalized adults, and both APS‐POQ‐R and SCQIPP have been used in postoperative inpatient settings. Therefore, the 24–48‐h period was considered appropriate for evaluating early postoperative pain outcomes and perceived quality of pain care among arthroplasty patients (Özdemir et al. 2022).

The researcher delivered both verbal and written explanations of the study, after which written informed consent was obtained from all participants. Patients were assured that their personal information would remain strictly confidential. Data collection took place either in patients' rooms or in designated areas of the orthopaedic and traumatology wards, and completion of the questionnaires required approximately 15–20 min.

### 2.4 Statistical Analysis

Data analysis was performed using IBM SPSS Statistics version 31.0 (IBM Corp., Armonk, NY, USA). The distribution of continuous variables was evaluated using skewness and kurtosis coefficients alongside visual inspection of histograms. Since APS‐POQ‐R and SCQIPP scores were approximately normally distributed, parametric statistical tests were applied. Descriptive statistics, including means, standard deviations, frequencies and percentages, were used to summarize sociodemographic and clinical characteristics of the participants. Independent samples *t*‐tests were employed to compare SCQIPP scores between groups of categorical variables. Associations between continuous clinical variables and APS‐POQ‐R and SCQIPP scores were examined using Pearson's correlation analysis. To identify factors associated with the quality of postoperative pain management, multiple linear regression analysis was conducted. Variables that were statistically significant in univariate analyses, together with those showing *p* < 0.10, were entered into the regression model. All model assumptions, including normality of residuals, linearity and absence of multicollinearity, were assessed and satisfied. A *p* value < 0.05 was considered statistically significant for all analyses. Because several subgroup comparisons and correlation analyses were conducted, these analyses were considered exploratory. No formal adjustment for multiple testing was applied; therefore, the findings from these analyses were interpreted cautiously, with emphasis placed on the main correlation analyses and the multivariable regression model.

### 2.5 Ethical Considerations

This study was conducted in accordance with the principles of the Declaration of Helsinki. Ethical approval was obtained from a non‐interventional clinical research ethics committee, and institutional permission was granted by the participating hospital administration before data collection. All participants were informed about the purpose of the study prior to data collection, and written informed consent was obtained from all participants.

## 3 Results

A total of 120 patients who underwent total knee and total hip arthroplasty, surgery were included in the analysis. The majority of participants were female (75%), and most had undergone total knee arthroplasty (81.7%). The mean total SCQIPP score was 50.77 ± 7.41, indicating a moderate‐to‐high perceived quality of postoperative pain care.

As shown in Table 1, SCQIPP scores differed significantly according to sex and surgical procedure. Female patients reported significantly higher SCQIPP scores compared with male patients (*p* = 0.036). Patients who underwent total knee arthroplasty surgery had higher SCQIPP scores than those who underwent total hip arthroplasty surgery (*p* = 0.014). No statistically significant differences in SCQIPP scores were observed according to type of surgery (emergency vs. elective), operated side, history of previous surgery, or previous surgery on the same site (*p* > 0.05). Patients who reported receiving information about pain management demonstrated significantly higher SCQIPP scores than those who did not receive such information (*p* < 0.001). Similarly, the use of non‐pharmacological pain management methods was associated with higher SCQIPP scores (*p* = 0.017). Higher SCQIPP scores were positively correlated with body mass index (*r* = 0.192, *p* = 0.036), initial pain score (*r* = 0.237, *p* = 0.009), and perceived usefulness of the information provided about pain treatment (*r* = 0.431, *p* < 0.001). No significant correlation was found between SCQIPP scores and age or current pain score (*p* > 0.05).

**TABLE 1:** Baseline comparison of patient characteristics by SCQIPP scores (*N* = 120).

Variable	SCQIPP	
	*N* (%)	Mean ± SD
Sex		
Female	90 (75)	51.47 ± 7.03
Male	30 (25)	48.67 ± 8.22
*p* value		0.036
Surgical procedure		
TKA	98 (81.7)	51.47 ± 7.33
THA	22 (18.3)	47.64 ± 7.10
*p* value		0.014
Type of surgery		
Emergency	24 (20)	49.42 ± 4.84
Elective	96 (80)	51.10 ± 7.91
*p* value		0.095
Operated side		
Right	72 (60)	51.33 ± 7.10
Left	48 (40)	49.92 ± 7.86
*p* value		0.153
History of previous surgery		
Yes	104 (86.7)	50.95 ± 7.23
No	16 (13.3)	49.56 ± 8.66
*p* value		0.244
Previous surgery on the same site		
Yes	23 (19.2)	53.04 ± 8.54
No	97 (80.8)	50.23 ± 7.06
*p* value		0.051
Received information about pain management		
Yes	51 (42.5)	53.88 ± 7.53
No	69 (57.5)	48.46 ± 6.46
*p* value		< 0.001
Use of non‐pharmacological pain management methods		
Yes	110 (91.7)	51.20 ± 7.46
No	10 (8.3)	46.00 ± 4.97
*p* value		0.017

Variable	Mean ± SD	Pearson correlation (PC)
Age	67.45 ± 11.92	0.011 (PC)
*p* value		0.907
BMI	28.82 ± 5.01	0.192 (PC)
*p* value		0.036
Initial pain score	5.88 ± 3.12	0.237 (PC)
*p* value		0.009
Current pain score	4.27 ± 2.84	0.141 (PC)
*p* value		0.124
Perceived usefulness of information provided about pain treatment	3.36 ± 4.15	0.431 (PC)
*p* value		< 0.001

Abbreviations: BMI, body mass index; PC, Pearson correlation; SCQIPP, Strategic and Clinical Quality Indicators in Postoperative Pain Management Questionnaire; THA, total hip arthroplasty; TKA, total knee arthroplasty.

Baseline comparisons of APS‐POQ‐R subscale scores according to sociodemographic and clinical characteristics are shown in Table 2. The highest mean APS‐POQ‐R subscale score was observed for pain severity and sleep interference, followed by perception of care. Female patients reported significantly higher adverse effect scores than male patients. Patients undergoing total hip arthroplasty reported higher activity interference, whereas patients undergoing total knee arthroplasty reported higher adverse effect scores. Receiving information about pain management and using non‐pharmacological methods were associated with higher scores in selected APS‐POQ‐R domains, particularly pain severity and sleep interference, adverse effects and perception of care. Correlation analyses indicated that higher initial pain scores were moderately correlated with pain severity and sleep interference (*r* = 0.526, *p* < 0.001) and perception of care (*r* = 0.433, *p* < 0.001). Current pain scores were also positively correlated with pain severity and sleep interference (*r* = 0.314, *p* < 0.001). Perceived usefulness of information provided about pain treatment showed a strong positive correlation with perception of care (*r* = 0.578, *p* < 0.001).

**TABLE 2:** Baseline comparison of patient characteristics by APS‐ POQ‐R subscales (*N* = 120).

Variable	Pain severity and sleep interference	Activity interference	Affective	Adverse effect	Perception of care
	Mean ± SD	Mean ± SD	Mean ± SD	Mean ± SD	Mean ± SD
Sex					
Female	30.29 ± 10.69	12.32 ± 5.51	12.13 ± 12.48	8.90 ± 7.66	18.72 ± 4.08
Male	31.80 ± 11.23	14.10 ± 5.55	10.70 ± 13.32	5.70 ± 5.65	17.60 ± 4.48
*p* value	0.255	0.064	0.297	0.009	0.103
Surgical procedure					
TKA	31.29 ± 10.54	12.22 ± 5.48	12.20 ± 12.51	8.63 ± 7.29	18.71 ± 4.09
THA	27.91 ± 11.75	15.18 ± 5.32	9.86 ± 13.40	5.73 ± 7.15	17.23 ± 4.51
*p* value	0.093	0.012	0.218	0.046	0.067
Type of surgery					
Emergency	33.50 ± 10.38	16.00 ± 3.58	12.08 ± 13.72	7.33 ± 8.06	19.08 ± 4.30
Elective	29.96 ± 10.84	11.96 ± 5.67	11.70 ± 12.45	8.29 ± 7.16	18.28 ± 4.17
*p* value	0.076	< 0.001	0.447	0.284	0.202
Operated side					
Right	30.47 ± 10.82	13.10 ± 5.21	12.92 ± 13.43	8.65 ± 7.84	18.57 ± 4.11
Left	30.96 ± 10.88	12.27 ± 6.04	10.06 ± 11.31	7.27 ± 6.46	18.25 ± 4.35
*p* value	0.405	0.213	0.106	0.147	0.342
History of previous surgery					
Yes	30.90 ± 10.54	12.75 ± 5.45	11.27 ± 11.94	8.36 ± 7.41	18.34 ± 4.25
No	29.13 ± 12.62	12.88 ± 6.36	15.06 ± 16.67	6.44 ± 6.70	19.13 ± 3.88
*p* value	0.271	0.467	0.196	0.166	0.243
Previous surgery on the same site					
Yes	35.39 ± 10.88	11.74 ± 4.62	18.09 ± 15.71	10.83 ± 9.57	20.78 ± 4.23
No	29.55 ± 10.52	13.01 ± 5.74	10.28 ± 11.40	7.45 ± 6.58	17.89 ± 4.01
*p* value	0.009	0.163	0.016	0.060	0.001
Received information about pain management					
Yes	34.69 ± 10.10	12.53 ± 5.17	12.53 ± 13.56	9.55 ± 7.98	20.84 ± 4.08
No	27.70 ± 10.39	12.94 ± 5.84	11.22 ± 12.01	7.03 ± 6.66	16.67 ± 3.32
*p* value	< 0.001	0.344	0.288	0.031	< 0.001
Use of non‐pharmacological pain management methods					
Yes	31.21 ± 10.76	12.53 ± 5.46	11.29 ± 12.01	8.44 ± 7.40	18.64 ± 4.29
No	24.70 ± 9.75	15.40 ± 6.11	17.10 ± 18.28	4.40 ± 5.34	16.30 ± 2.06
*p* value	0.034	0.059	0.174	0.048	0.004

Variable	Pearson correlation (PC)	Pearson correlation (PC)	Pearson correlation (PC)	Pearson correlation (PC)	Pearson correlation (PC)
Age	−0.079 (PC)	0.124 (PC)	−0.027 (PC)	−0.049 (PC)	−0.042 (PC)
*p* value	0.390	0.178	0.771	0.596	0.651
BMI	0.042 (PC)	−0.154 (PC)	0.115 (PC)	0.044 (PC)	0.193 (PC)
*p* value	0.652	0.093	0.213	0.633	0.035
Initial pain score	0.526 (PC)	0.171 (PC)	0.047 (PC)	0.205 (PC)	0.433 (PC)
*p* value	< 0.001	0.062	0.611	0.025	< 0.001
Current pain score	0.314 (PC)	0.174 (PC)	0.157 (PC)	0.088 (PC)	0.169 (PC)
*p* value	< 0.001	0.058	0.087	0.341	0.066
Perceived usefulness of information provided about pain treatment	0.338 (PC)	−0.042 (PC)	0.085 (PC)	0.160 (PC)	0.578 (PC)
*p* value	< 0.001	0.646	0.357	0.081	< 0.001

Abbreviations: APS‐POQ‐R, The American Pain Society Patient Outcome Questionnaire; BMI, body mass index; PC, Pearson correlation; THA, total hip arthroplasty; TKA, total knee arthroplasty.

Correlations between APS‐POQ‐R subscales and SCQIPP scores are presented in Table 3. Among the APS‐POQ‐R subscales, only the perception of care subscale demonstrated a statistically significant positive correlation with SCQIPP scores (*r* = 0.521, *p* < 0.001). No significant correlations were observed between SCQIPP scores and pain severity, sleep interference, activity interference, affective responses or adverse effects (*p* > 0.05).

**TABLE 3:** Descriptive statistics and correlations between APS‐POQ‐R subscales and SCQIPP total score (*n* = 120).

Variable	Mean ± SD	*r*	*p*
Pain severity and sleep interference	30.67 ± 10.80	0.094	0.310
Activity interference	12.77 ± 5.55	−0.005	0.953
Affective	11.78 ± 12.65	0.005	0.961
Adverse effect	8.10 ± 7.32	0.103	0.262
Perception of care	18.44 ± 4.19	0.521	< 0.001
SCQIPP	50.77 ± 7.41	1	—

Abbreviations: APS‐POQ‐R, The American Pain Society Patient Outcome Questionnaire; SCQIPP, Strategic and Clinical Quality Indicators in Postoperative Pain Management Questionnaire.

Multiple linear regression analysis was conducted to identify factors independently associated with SCQIPP scores (Table 4). The overall model was statistically significant, *F*(2,117) = 24.710, *p* < 0.001, and explained approximately 29.7% of the variance in SCQIPP scores (*R* ^2^ = 0.297). Perception of care emerged as the strongest independent determinant of SCQIPP scores (B = 0.72, 95% CI: 0.39, 1.05, *p* < 0.001). In addition, perceived usefulness of the information provided about pain treatment was independently associated with higher SCQIPP scores (B = 0.35, 95% CI: 0.01, 0.69, *p* = 0.042).

**TABLE 4:** Factors associated with perceived quality of postoperative pain care following total hip or total knee arthroplasty based on multiple regression analysis (*n* = 120).

Independent variable	B (95% CI)	SE	*p*
Intercept	36.29 (30.62, 41.96)	2.86	< 0.001
Perception of care	0.72 (0.39, 1.05)	0.17	< 0.001
Perceived usefulness of information provided about pain treatment	0.35 (0.01, 0.69)	0.17	0.042

*Note:* *R* = 0.545. *R* ^2^ = 0.297. Adjusted *R* ^2^ = 0.285.

Abbreviations: B, regression coefficient; CI, confidence interval; SCQIPP, Strategic and Clinical Quality Indicators in Postoperative Pain Management Questionnaire; SE, standard error.

## 4 Discussion

This study examined perceived quality of postoperative pain care and patient‐reported pain‐related outcomes among patients undergoing total hip or total knee arthroplasty. The main findings were that patients reported a moderate‐to‐high perceived quality of postoperative pain care, and that pain severity and sleep interference represented important patient‐reported pain‐related outcomes during the early postoperative period. In the comparative analyses, SCQIPP scores differed according to sex, surgical procedure, receipt of pain‐management information and use of non‐pharmacological pain management methods. Among APS‐POQ‐R domains, perception of care was the only subscale significantly correlated with SCQIPP scores. In multivariable analysis, perception of care and perceived usefulness of pain‐related information were the only independent determinants of higher perceived quality of postoperative pain care. These findings suggest that postoperative pain care should be evaluated not only through pain intensity or symptom burden, but also through patients' perceptions of how pain care is communicated, delivered and experienced.

With respect to patient characteristics, female patients reported higher SCQIPP scores than male patients, whereas women also had higher adverse‐effect scores on the APS‐POQ‐R. These findings may indicate that perceptions of pain care quality are influenced not only by symptom burden, but also by differences in expectations, reporting patterns, communication and clinician responsiveness. A recent systematic review of postoperative pain and pain management found that female sex was frequently associated with higher postoperative pain scores and differences in analgesic management, factors that may influence both care needs and patients' perceptions of care (Thurston et al. 2023). In the present study, however, female patients still rated pain care more positively, suggesting that higher symptom burden does not necessarily translate into poorer perceptions of care when communication and support are adequate.

Patients undergoing total knee replacement reported higher SCQIPP scores than those undergoing total hip replacement, whereas patients undergoing hip replacement reported greater activity interference. These procedure‐related differences may reflect variation in perioperative pathways, rehabilitation demands, mobility restrictions and patient expectations across arthroplasty procedures. Recent arthroplasty literature suggests that postoperative experience is shaped not only by pain intensity itself but also by how patients perceive the overall recovery process, including whether perioperative care meets expectations and supports rehabilitation effectively (Bric et al. 2024; Mooiweer et al. 2024; Halvorson et al. 2024). In fast‐track and outpatient arthroplasty pathways, patients' evaluations of recovery are closely associated with the information they receive, their readiness for discharge and the support available during the early postoperative period (Bric et al. 2024; Hofstad et al. 2024; van Egmond et al. 2024). Taken together, these findings suggest that procedure‐specific pain education, expectation management and recovery guidance may be important for optimizing patients' perceptions of care quality after arthroplasty.

The association between previous surgery on the same site and higher pain severity, greater sleep interference and more pronounced affective responses is clinically plausible and may indicate a more complex postoperative pain profile in these patients. Recent evidence suggests that a history of prior surgery, particularly before knee arthroplasty, is associated with less favourable postoperative outcomes, including higher risks of persistent pain and stiffness, implying that repeated surgical exposure may mark a subgroup with greater biological and psychosocial vulnerability rather than simply greater familiarity with the recovery process (Li et al. 2025). Contemporary arthroplasty research also increasingly shows that postoperative pain experience is shaped by factors such as expectations and sleep disturbance, all of which may amplify symptom reporting and recovery burden (Kris et al. 2025; Dandamudi et al. 2025). In this context, previous surgery may be better understood as a marker of a more complicated pain and recovery trajectory, rather than as an experience that necessarily improves patients' appraisal of care. However, the relationship between prior surgery at the same site and patient‐rated care quality remains underexplored in recent arthroplasty literature and warrants further investigation.

Patients who received pain‐management information reported higher SCQIPP scores. This finding suggests that education may contribute positively to patients' evaluations of postoperative pain care. A recent systematic review and meta‐analysis concluded that preoperative education can improve short‐term postoperative pain control in orthopaedic surgery, although its effects on longer‐term functional recovery appear less consistent (Wang et al. 2024). Likewise, a randomized controlled trial in total hip arthroplasty found that structured preoperative education improved clinical outcomes, length of stay, health‐related quality of life, and psychological outcomes after surgery (Amprachim et al. 2024). Taken together, these findings suggest that education may improve patients' evaluations of postoperative pain care by enhancing preparedness, shaping expectations, and increasing confidence in pain self‐management during recovery (Amprachim et al. 2024).

Patients who used non‐pharmacological pain management methods also reported higher SCQIPP scores. This aligns with the increasing emphasis on multimodal postoperative pain management, in which non‐pharmacological strategies are used to complement analgesic medication (El‐Boghdadly et al. 2024). A recent review of non‐pharmacological postoperative pain interventions concluded that approaches such as preoperative education, physical methods, and mind–body strategies can reduce pain intensity, decrease opioid use, and improve satisfaction and recovery outcomes, while also highlighting the key role of nurses and other frontline professionals in implementing these methods in practice (Niyonkuru et al. 2025). Although the current study cannot determine which specific non‐pharmacological strategies were most effective, the findings suggest that patients may perceive pain care more positively when they are offered practical, supportive options in addition to medication.

Interestingly, receiving information about pain management and using non‐pharmacological methods were also associated with higher pain severity and sleep interference, and higher adverse‐effect scores on the APS‐POQ‐R. Although this finding may appear contradictory, several explanations are possible. Patients with greater symptom burden may attract more clinical attention and therefore receive more counselling and more encouragement to try additional coping strategies (Schwenkglenks et al. 2014). It is also possible that patients experiencing more intense pain become more attentive to the information they receive and more likely to recall or use adjunctive measures. This interpretation is compatible with the multidimensional nature of pain outcomes reported in postoperative pain literature, where symptom severity and satisfaction or care appraisal do not always move in the same direction (Jiang et al. 2022; Schwenkglenks et al. 2014). Because the present study was cross‐sectional, these relationships should be interpreted cautiously; however, they may suggest that pain education is delivered more reactively to patients with greater symptom burden.

The finding that perception of care was the only APS‐POQ‐R subscale significantly associated with SCQIPP is clinically important. It suggests that patients may distinguish between the severity of postoperative pain and their evaluation of whether pain care was responsive, supportive and appropriate. Therefore, perceived quality of pain care should not be interpreted only as a reflection of pain intensity or symptom burden. Rather, it appears to be shaped by how care is communicated and delivered, including whether patients feel listened to, informed and supported during the early postoperative period. This interpretation is consistent with findings from the international PAIN OUT registry, where postoperative satisfaction was associated with patients' actual pain experience, but more strongly with their impressions of improvement and the appropriateness of care, including information provision and involvement in treatment decisions (Schwenkglenks et al. 2014). Similarly, a recent orthopaedic observational study reported that satisfaction with postoperative pain management was higher when patients received information about pain‐treatment options and used non‐pharmacological methods, even though high pain levels were still common early after surgery (Khalil et al. 2025).

The regression analysis provided the strongest synthesis of factors independently associated with perceived quality of postoperative pain care. Perception of care was the strongest independent determinant of SCQIPP scores, and perceived usefulness of pain‐related information was also independently associated with higher SCQIPP scores. These findings highlight the importance of patient‐centred communication and meaningful education. Information must not only be provided, but also be understandable, relevant, and useful from the patient's perspective. A recent mixed‐methods systematic review on acute pain education found that patients particularly value education that explains what pain to expect, how analgesics should be used, and how adverse effects should be managed, and emphasized that education should be embedded in communication and partnership with patients and caregivers (Bérubé et al. 2024). This is especially relevant during orthopaedic recovery, when patients must rapidly translate information into action during mobilization, exercise, and self‐management in the immediate postoperative period.

The findings have several implications for nursing and multidisciplinary postoperative care. Quality improvement efforts may benefit from targeting care experience domains, including timely response to pain reports, communication, and shared decision‐making. In addition, pain education should be structured to maximize perceived usefulness, potentially by using brief, patient‐friendly materials, teach‐back methods, and individualized counselling that addresses what patients find most relevant. These educational strategies are recommended because the perceived usefulness of pain‐related information was independently associated with higher SCQIPP scores in the present study, suggesting that the quality, clarity, and relevance of education may influence patients' evaluations of postoperative pain care. Moreover, integrating non‐pharmacological strategies into routine care, while actively encouraging and guiding their use, may strengthen patients' appraisal of pain management quality. These results suggest that improving perceived quality of pain care may require not only effective symptom control but also high‐quality communication and education, with particular attention to how patients appraise the care they receive.

## 5 Limitations

Several limitations should be considered when interpreting the findings. First, the cross‐sectional design limits causal interpretation of the observed associations. Second, data were collected in a single public hospital, which may restrict generalizability. Third, although patients were recruited during the study period using consecutive convenience sampling, the total number of patients screened, the number who declined participation and reasons for non‐participation were not systematically recorded. Therefore, potential selection bias cannot be excluded. Fourth, patient‐reported measures may have been influenced by expectations, response tendencies and recall, particularly for education and non‐pharmacological strategies. In addition, several subgroup comparisons and correlation analyses were conducted without formal adjustment for multiple testing; therefore, some statistically significant findings should be interpreted cautiously. Finally, the study included only patients undergoing total hip or total knee arthroplasty; therefore, the findings may not be generalisable to all types of major orthopaedic surgery.

## 6 Recommendations for Further Research

Future research should use longitudinal and multicentre designs to clarify the temporal relationship between perceived quality of postoperative pain care and patient‐reported outcomes. Interventional studies are also needed to test whether structured educational approaches, teach‐back strategies, and nurse‐led support for non‐pharmacological pain management improve both perceived care quality and recovery‐related outcomes after arthroplasty. Qualitative studies may also help identify which aspects of communication, education, and support are most meaningful to patients during early postoperative recovery.

### 6.1 Implications for Policy and Practice

These findings suggest that postoperative pain management should be approached as both a symptom‐control issue and a care‐quality issue. Nurses and multidisciplinary teams should routinely assess patients' perceptions of care, provide tailored and understandable pain‐related information, and actively support non‐pharmacological pain management strategies. Clinical policies and postoperative care pathways should therefore incorporate patient‐centred communication, education and shared decision‐making as core components of quality pain care in orthopaedic practice.

## 7 Conclusion

In this cohort of patients undergoing total hip or total knee arthroplasty, the perceived quality of postoperative pain care was most strongly associated with patients' perception of care and the perceived usefulness of pain‐related information. These findings emphasize that improving postoperative pain management may require not only effective analgesic treatment but also meaningful patient‐centred communication, useful education and support for non‐pharmacological strategies.

## Author Contributions

S.S. developed the study concept and design. D.T., E.N.Ç., and S.K. conducted data collection, while S.S. performed the statistical analyses. S.S., D.T., E.N.Ç., and S.K. jointly contributed to the analysis and interpretation of the data. S.S. also supervised the project and coordinated all study activities. All authors participated in drafting or critically revising the manuscript and share responsibility for the accuracy, integrity, and overall quality of the work.

## Funding

The authors have nothing to report.

## Disclosure

All authors take responsibility for all aspects of the reliability and freedom from bias of the data presented and their discussed interpretation.

## Ethics Statement

The study adhered to the ethical standards outlined in the Declaration of Helsinki. Approval for the research was obtained from the Usak University Non‐Interventional Clinical Research Ethics Committee (04 April 2024; Decision No: 362–362‐20), and permission to conduct the study was granted by the hospital administration (25 December 2023; Approval No: E‐45786011‐599‐232,485,867).

## Consent

All participants provided written informed consent before participating in the study.

## Conflicts of Interest

The authors declare no conflicts of interest.

## Supporting information


**Data S1:** jocn70443‐sup‐0001‐DataS1.pdf.

## Data Availability

The data that support the findings of this study are available from the corresponding author upon reasonable request.
